# Diversity and distribution of *Laonice* species (Annelida: Spionidae) in the tropical North Atlantic and Puerto Rico Trench

**DOI:** 10.1038/s41598-019-45807-7

**Published:** 2019-06-25

**Authors:** Theresa Guggolz, Karin Meißner, Martin Schwentner, Angelika Brandt

**Affiliations:** 1 Zoological Museum Hamburg, Center of Natural History, Universität Hamburg, Martin-Luther-King-Platz 3, D-20146 Hamburg, Germany; 2 German Centre for Marine Biodiversity Research, Senckenberg am Meer, c/o Universität Hamburg, Martin-Luther-King-Platz 3, D-20146 Hamburg, Germany; 3 Senckenberg Naturmuseum, Senckenberganlage 25, 60325 Frankfurt, Germany

**Keywords:** Biodiversity, Population genetics

## Abstract

*Laonice* Malmgren, 1867 (Annelida: Spionidae) is a common polychaete genus in the deep-sea. Although most species are quite well studied morphologically, fragmentation and other damage that occurs during sampling often hampers morphological species identification of deep-sea specimens. In this study, we employ three molecular markers (16S, COI and 18S) to study the biodiversity and the distribution patterns of *Laonice* from the tropical North Atlantic and the Puerto Rico Trench. Based upon different molecular analyses (Automated Barcode Gap Discovery, pairwise genetic distances, phylogenetics, haplotype networks) we were able to identify and differentiate eight *Laonice* species. Up to four of these species co-occurred sympatrically at the same station. The majority of species were found at multiple stations and two species in the eastern as well as western Atlantic had ranges of up to 4,000 km. Genetic differentiation across these extensive geographic distances was very low. Surprisingly, one 16S haplotype was shared between individuals 2,776 km apart and individuals from the Caribbean and the abyssal plain in the eastern Atlantic (>3,389 km) differed in only a single mutation in 16S. Our results suggest that members of this genus successfully disperse across large geographic distances and are largely unaffected by topographic barriers.

## Introduction

Spionidae Grube, 1850^1^ is one of the most abundant and diverse groups of polychaetes and occur in almost all marine habitats, from shallow waters to the deep-sea^2^. All spionids are characterized by a pair of long palps, used for deposit or suspension feeding; most species are tube-dwellers, but free-living or commensal species are also found within the taxon^3,4^. Like several other annelid taxa, Spionidae are soft-bodied and very fragile and are, therefore, rarely found undamaged in deep-sea samples. These incomplete and fragmented individuals often lack crucial taxonomic characters, hampering their identification^5^. Nonetheless, the spionid genus *Laonice* Malmgren, 1867^6^ is well studied, especially species from the deep sea of the North Atlantic^7–10^. To facilitate the identification of *Laonice* species extensive studies on species-specific characters were conducted and four subgenera were suggested based on morphological characters^8,11^. However, the recently published first molecular phylogenetic study on *Laonice* rejected two of these four subgenera^5^. Several *Laonice* species have been reported from a wide geographical range, and the presumed long planktonic life and planktotrophic larvae would offer the potential for long-distance dispersal^12–14^. However, *Laonice cirrata* (Sars, 1851^15^), a presumed widespread species, was shown to probably represent several geographically restricted species^5,16^.

The abyssal Atlantic Ocean is divided by the Mid-Atlantic Ridge (MAR) longitudinally into eastern and western basins^17^. Due to its geology, the MAR is believed to represent a dispersal barrier for some components of the abyssal benthic fauna^18–21^. However, the MAR is not a closed barrier as several Fracture Zones interrupt it. When two tectonic plates passing each other in parallel to their original motions, a so-called transform fault is formed at the offsets of the ridge^22^. Over geological time the movement results in an extension past the transform fault in opposite directions, the Fracture-Zones^23^.

Our study area encompasses the abyssal eastern and western basins in the tropical North Atlantic along the Vema Fracture Zone as well as the Puerto Rico Trench. The first morphological studies rejected a barrier effect of the MAR on the distribution of selected widespread spionid species in the abyss of the tropical North Atlantic, though other species were found to be limited to either side of the MAR^24^. However, the presence of morphologically cryptic species could not be ruled out.

The aim of this study is to investigate the diversity and distribution of *Laonice* from the tropical North Atlantic and the Puerto Rico Trench with molecular tools and further assess the potential barrier effect of the MAR on abyssal spionid taxa.

## Material and Methods

### Collection and identification of specimens

**Figure 1:**
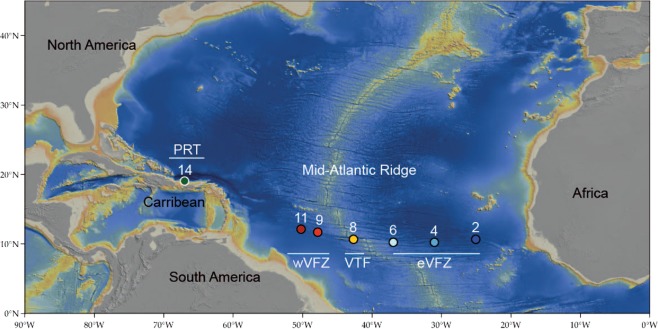
Map of Vema and PRT (modified after a map of N. Augustin).

All analysed specimens were collected from the tropical North Atlantic and the Puerto Rico Trench during the VEMA-Transit expedition in December 2014–January 2015 (Fig. 1, Supplement 1). Sampling was conducted with a camera-equipped epibenthic sledge at depths between 4918–5736 m, followed by a fixation of either cooled 96% ethanol or 4% buffered formalin. More detailed information about sample treatment and sampling localities are described in Guggolz *et al*.^24^ and Devey *et al*.^25^. According to the geographical position, four areas were defined as following: the eastern part of the Vema-Fracture Zone (eVFZ), extending eastwards from the MAR in the Cape Verde Basin; the western part of the Vema Fracture Zone (wVFZ), extending westwards from the MAR in the Demerara Basin; the Vema Transform Fault (VTF), located between these two areas in the MAR; the Puerto Rico Trench (PRT), located in the shallower part of the trench near Puerto Rico (Fig. 1). Distances between areas varied between 276 km (wVFZ) and 1,298 km (eVFZ). The eastern-most and western-most studied sites were separated by 4,610 km (Table 1).

**Table 1:** Distances (in km) between collection localities. Areas: eastern Vema Fracture Zone (eVFZ), western Vema-Fracture Zone (wVFZ), Vema Transform Fault (VTF), Puerto Rico Trench (PRT).

Area	eVFZ			VTF	wVFZ		PRT
Site	2	4	6	8	9	11	14
2	0						
4	659	0					
6	1,298	640	0				
8	1,925	1,269	630	0			
9	2,503	1,851	1,216	589	0		
11	2,776	2,125	1,492	865	276	0	
14	4,610	3,992	3,389	2,788	2,213	1,946	0

All specimens were sorted and identified at least to genus level using stereo zoom and compound microscopes. All specimens identified as *Laonice* and aff. *Lindaspio*^24^ were analysed. The identification of the latter has been revised and reassigned to *Laonice* (unpublished data). Specimens have been deposited in the collection of the Center of Natural History (Universität Hamburg, Germany) (Supplement 1).

### DNA extraction, PCR amplification, sequencing and alignment

DNA was extracted with Chelex 100. Depending on the size of specimens, one or two parapodia were dissected and transferred into 30 µl of 10% Chelex solution in purified water and incubated for 30 minutes at 56 °C and 10 minutes at 99 °C. Polymerase Chain Reactions (PCR) were performed with a total volume of 15 µl consisting of 1.5 µl DNA extract, 7.5 µl AccuStart II PCR ToughMix (Quanta Bio, Germany), 0.6 µl of each primer (10mmol), 0.3 µl of GelTrack loading dye (QuantaBio, Germany) and 4.8 µl Millipore H_2_O. Fragments of mitochondrial (16S and COI) and nuclear (18S) rRNA genes were amplified (see Table 2 for list of all primers). PCR amplification had an initial denaturation step of 94 °C for 3 min, followed by 35 cycles of 30 s at 94 °C, 45 s at 43 °C and 45 sec at 72 °C, followed by a final elongation step for 5 min at 72 °C. Success of amplification was determined via gel electrophoresis on 1% agarose/TAE gel. For sequencing, 8 µl of the PCR products were purified using FastAP (1.6 µl; 1 U/µl) and Exonuclease I (0.8 µl; 20 U/µl) (Thermo Fisher Scientific, Germany) with an incubation time of 37 °C for 15 min followed by 15 min with 85 °C and a final holding temperature of 14 °C. Purified PCR products were sent to Macrogen Europe, Inc. (Amsterdam-Zuidoost, Netherlands) for sequencing. All in all, 80 specimens were successfully sequenced for 16S, a subset of 27 specimens for COI and 47 specimens for 18S. Sequences were assembled and corrected with Geneious 6.1.8 (http://www.geneious.com)^26^ and all sequences were deposited in GenBank (for accession numbers see Supplement 1). The obtained sequences of the different gene fragments were aligned separately using MUSCLE^27^ implemented in Genious 6.1.8.

**Table 2:** All Primers used in this study.

Gene	Primer	Primer sequence 5′-3′	Authors
COI	jgLCO1490	TNTCNACNAAYCAYAARGAYATTGG	Geller *et al*.^65^
	jgHCO2198	TANACYTCNGGRTGNCCRAARAAYCA	Geller *et al*.^65^
	LCO1490	GGTCAACAAATCATAAAGATATTG	Folmer *et al*.^66^
	HCO2198	TAAACTTCAGGGTGACCAAAAAATCA	Folmer *et al*.^66^
	LCO2	TCNACHAAYCATAAAGAYATTGGAAC	Designed by L. Krebes and R. Bastrop
	HCOout	CCAGGTAAAATTAAAATATAAACTTC	Carpenter & Wheeler^67^
16S	16Sar	CGCCTGTTTATCAAAAACAT	Palumbi^68^
	16Sbr	CCGGTCTGAACTCAGATCACGT	Palumbi^68^
	16Sb-L	CCGGTCTGAACTCAGATCACGT	Palumbi *et al*.^69^
18S	Uni 18SF	GCTTGTCTCAGAGATTAAGCC	Dzikowski *et al*.^70^
	HET 18SR	ACGGAAACCTTGTTACGA	Dzikowski *et al*.^70^

### Initial identification of species, phylogenetic analyses and haplotype networks

To obtain a first estimation of the number of species among *Laonice* investigated, the Automated Barcode Gap Discovery (ABGD^28^) was conducted separately for each of the three genes (16S, COI, 18S). The ABGD identifies potential barcoding gaps separating hypothetical species, based on the assumption that interspecific genetic distances are larger than intraspecific distances. The ABGD analysis was run on the web-based version of the software (http://wwwabi.snv.jussieu.fr/public/abgd/abgdweb.html), using uncorrected p distances (Table 3), which were calculated with MEGA7^29^ on all available sequences. Standard settings were kept, except for Pmin (0.005), the numbers of steps (100) and the relative gap width (X = 0.5).

**Table 3:** Percentage of uncorrected p-distances within and among lineages for COI, 16S and 18S (see upper right corner). “X” means no or only one sequence available.

	*Laonice* sp. A	*Laonice* sp. B	*Laonice* sp. C	*Laonice* sp. D	*Laonice* sp. E	*Laonice* sp. F	*Laonice* sp. G	*Laonice* sp. H
*Laonice* sp. A	0 X 0.1							16S COI 18S
*Laonice* sp. B	18.7–19.9 21.9 0.9–1.3	0.4 0.0 0.3						
*Laonice* sp. C	16.2 23.0 0.7–0.8	10.7 17.4 0.3–0.6	X X X					
*Laonice* sp. D	17.0–23.0 21.7–22.6 1.9–2.1	16.1–20.4 20.3–20.7 1.4–1.8	15.8–21.4 20.2–20.7 1.3	0.0–1.4 0.0–0.7 0.0				
*Laonice* sp. E	17.2 X 2.0–2.1	14.8–15.3 X 1.4–1.8	14.7 X 1.3	6.3–8.2 X 0.1	X X X			
*Laonice* sp. F	17.5–19.4 23.0 1.8–1.9	14.8–17.0 20.4–20.5 1.3–1.6	15.0–17.3 20.2 1.2	8.2–12.7 14.0–14.8 0.1–0.2	7.1–8.2 X 0.2	0.0–1.7 X 0.0		
*Laonice* sp. G	18.5–21.9 21.9–22.0 1.8–1.9	15.3–17.1 20.2–20.7 1.3–1.6	15.1–15.5 20.5–20.7 1.2	8.2–10.8 15.3–15.9 0.1–0.2	4.7–5.1 X 0.2	2.8–4.0 8.6–8.8 0.0	0.0–0.2 0.5 0.0	
*Laonice* sp. H	18.5–19.7 22.3–22.7 2.0–2.8	15.8–16.4 22.4–22.9 1.5–2.5	15.4–17.1 20.7–21.0 1.4–2.1	4.1–8.2 11.7–12.4 0.1–0.9	7.1–8.2 X 0.2–0.7	10.9–13.2 17.0–17.5 0.2–0.9	6.4–7.3 14.2 –14.9 0.2–0.9	0.0–1.0 0.2–0.7 0.0

To assess the phylogenetic relationships among the studied specimens and to assess whether the lineages suggested by ABGD are monophyletic, phylogenetic analyses were performed with Bayesian inference. All three gene fragments were analysed separately and concatenated with MrBayes (version 3.2^30^) online with CIPRES Science Gateway V.3.3 (www.phylo.org)^31^. For the analyses of the 16S and COI genes, *Marenzelleria neglecta* Sikorski & Bick, 2004^32^, *Malacoceros indicus* (Fauvel, 1928)^33^, *Polydora hoplura* Claparède, 1868^34^ and *Spio blakei* Maciolek, 1990^35^ were employed as outgroups (Supplement 2). Four chains were run for 10^7^ generations, with sampling every 1200^th^ generation, and discarding the first 25% as burn-in. The GTR + I + G substitution model was identified by MEGA7 as the best fitting model under the AIC criterion.

Guggolz *et al*.^24^ studied the same *Laonice* individuals morphologically. That data was used to identify morphological differences between the herein delimited species and to add another line of evidence for species delimitation.

To assess the genus-wide phylogenetic relationships of the herein studied *Laonice* and to find out whether any of these species have a wider distribution than anticipated by our own data, a phylogenetic analysis with *Laonice* sequences available from GenBank was conducted for COI and 16S. Additional data includes: *Laonice* from expeditions around Iceland (IceAGE I + II^5^) and other GenBank entries^14,36–43^ (Supplement 2). The genus-wide analysis was focussed on the COI data, because of the more comprehensive COI data being available (Supplement 2), even if analysis with 16S data was also conducted (Supplement 3).

To better visualize the geographic distribution of the genetic diversity median-joining haplotype networks were generated with Network 5.0.0.3^44^ (http://fluxus-engineering.com/) for each gene fragment. The generated haplotype networks were redrawn with Adobe Illustrator CS6.

Analyses of population differentiation were performed with Arlequin 3.5^45^ for species with sufficiently large specimen numbers (at least four specimens per site). Pairwise Φst was calculated for *Laonice* sp. D, F, H (16S). For *Laonice* sp. D areas eVFZ and wVFZ, for *Laonice* sp. F areas eVFZ and VTF and for *Laonice* sp. H the areas eVFZ, wVFZ and VTF were compared (Tables 4 and 5).

**Table 4:** Population indices for 16S of selected *Laonice* species among sites and geographic areas. Nucleotide diversity, Tajima’s D and Fu’s Fs are reported only for the areas, not the individual sites. (eVFZ: eastern Vema Fracture Zone, wVFZ: western Vema-Fracture Zone, VTF: Vema Transform Fault).

	site	No. of ind.	No. of haplotypes	Nucleotide diversity ± SD	Tajima’s D (p-value)	Fu’s F_s_ (p-value)
***Laonice*** **sp**. **D**						
eVFZ	2	18	5	0.0035 ± 0.0027	−0.465 (0.601)	−0.679 (0.2630)
	4	8	6			
	6	4	1			
wVFZ	9	3	2	0.0018 ± 0.0023		
***Laonice*** **sp**. **F**						
eVFZ	2	5	5	0.0084 ± 0.0055	−1.174 (0.089)	−1.205 (0.098)
	4	3	3			
VTF	8	5	5	0.00187 ± 0.0019		
***Laonice*** **sp**. **H**						
eVFZ	6	3	3	0.0025 ± 0.0022	−0.333 (0.465)	0.261 (0.425)
VTF	8	3	6	0.0021 ± 0.0020		
wVFZ	9	2	4	0.0024 ± 0.0024

**Table 5:** Pairwise Φst values among different sites for 16S of selected *Laonice* species among sites. (eVFZ: eastern Vema Fracture Zone, wVFZ: western Vema-Fracture Zone, VTF: Vema Transform Fault).

*Laonice* sp. D	eVFZ	site 4	site 6	wVFZ
	site 2			site 9
site 2	0.000			
site 4	0.000	0.000		
site 6	0.175	0.111	0.000	
site 9	0.000	0.000	0.015	0.000
***Laonice*** **sp**. **F**	**eVFZ**	**eVFZ**	**VTF**	
	**site 2**	**site 4**	**site 8**	
site 2	0.000			
site 4	0.000	0.000		
site 8	0.000	0.008	0.000	
***Laonice*** **sp**. **H**	**eVFZ**	**VTF**	**wVFZ**	
	**site 6**	**site 8**	**site 9**	
site 6	0.000			
site 8	0.000	0.000		
site 9	0.000	0.000	0.000	

## Results

### Alignment

The alignment of the 16S fragment included a total of 79 sequences with a length of 525 bp, of which 223 bp were variable and 165 bp were parsimony informative. The COI alignment featured 26 sequences and had a length of 694 bp, of which 283 bp were variable and 207 bp parsimony informative. The alignment contained no indels and the derived amino acid alignment consisted of 208 amino acids, with 16 variable amino acids and no stop codons. The genus-wide COI alignment featured 134 sequences (including outgroup) and had a length of 683 bp, of which 324 were variable and 301 were parsimony informative. The alignment of the 18S fragment consisted of 46 sequences with 2195 bp, of which only 68 bp were variable and 34 bp were parsimony informative.

### Species delimitation

The ABGD analysis of the 16S dataset retrieved eight main lineages when barcode thresholds of 0.5–4.5% were employed. For now, we use the term lineages rather than species, as not all of them necessarily correspond to species. To the eight lineages, we will refer to as *Laonice* sp. A–H. With higher threshold values several lineages collapsed (4.6–6.5% = 3 lineages), or all lineages collapsed into a single lineage (>6.7%). The analysis of the COI dataset resulted in seven lineages (barcode thresholds 0.5–10%). The seven lineages identified with COI are in full agreement with the lineages derived with 16S, with the same specimens being clustered together. The discrepancy between 16S and COI is due to the absence of one lineage, *Laonice* sp. E (PVT 471_I), which was not successfully sequenced for COI. Pairwise genetic distances (uncorrected *p*-distances) between the lineages ranged for 16S from 2.8–23%, for COI from 8.6–23% and for 18S from 0–2.8% (based on the eight lineages derived by 16S) (Table 3). The lowest pairwise distances were found between the lineages F and G (16S: 2.8–4%; COI: 8.6–8.8%; 18S: 0%), whereas all other pairwise distances between the lineages were higher than 4.1% for 16S, 12.2% for COI and 0.1% for 18S. Within lineages, the highest observed pairwise distances were 1.7% for 16S, 0.7% for COI and 0.3% for 18S (Table 3).

**Figure 2:**
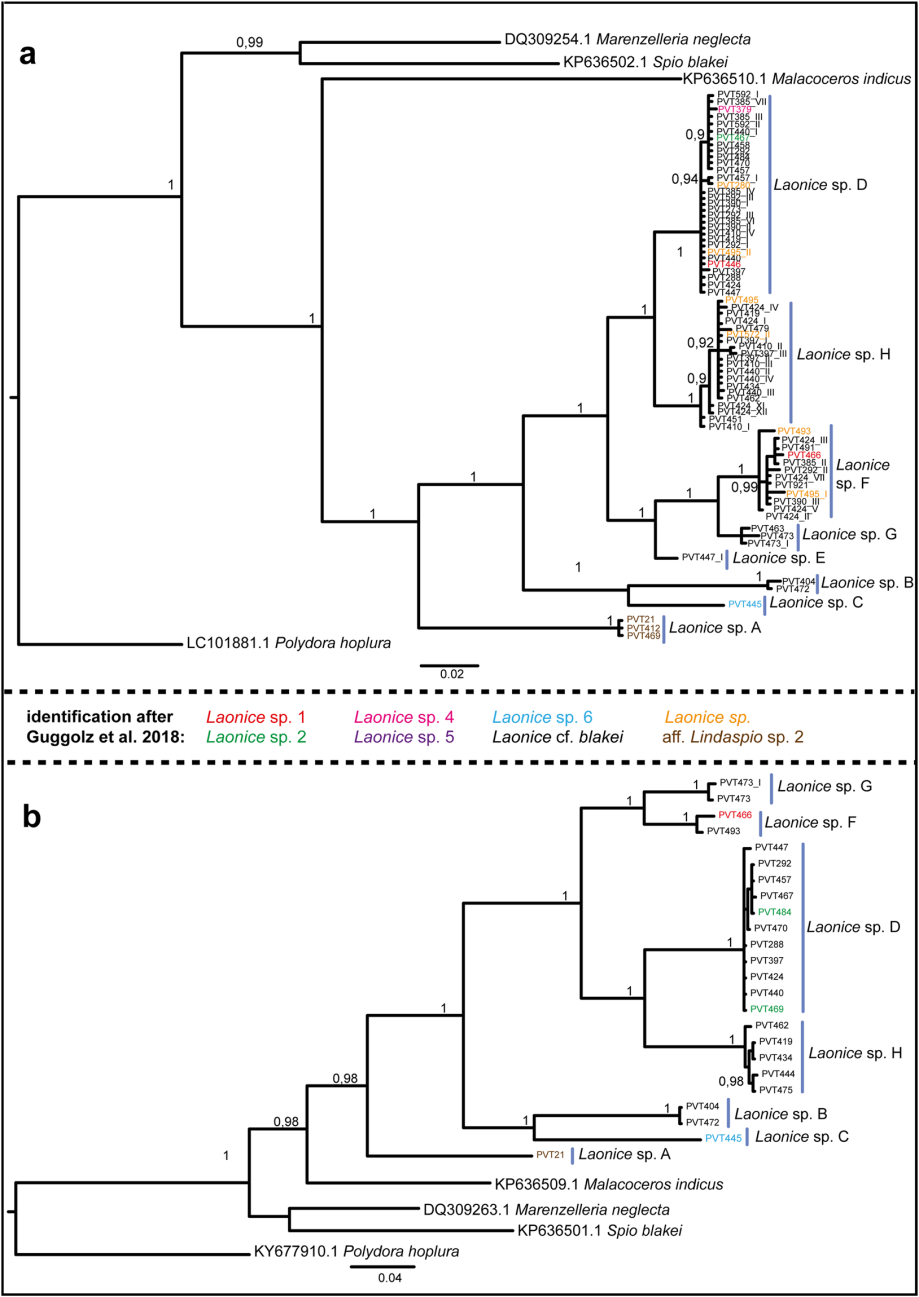
Phylogenetic tree of Laonice specimens from the Vema-Transit expedition based on mitochondrial 16S (**a**) and COI (**b**) gene fragments. Posterior probabilities shown next to the nodes (values below 0.8 are not shown). Morphological identification after Guggolz *et al*. 2018^24^ are color coded (see legend in the middle).

The phylogenetic analyses of COI and 16S recovered lineages A-H as reciprocal monophyletic with full support each (Fig. 2). Also the phylogenetic relationships among the lineages were very similar for 16S and COI. *Laonice* sp. F and G are sister species (in 16S, *Laonice* sp. E clusters with these two species), as are *Laonice* sp. C and B as well as *Laonice* sp. D and H. Differences between the analyses of the 16S and COI data are found in the position of *Laonice* sp. A. In COI, *Laonice* sp. A is found to be a sister taxon to *Laonice* sp. B and C (Fig. 2a), whereas in 16S *Laonice* sp. A is placed as a sister taxa to all other species (Fig. 2b).

**Figure 3:**
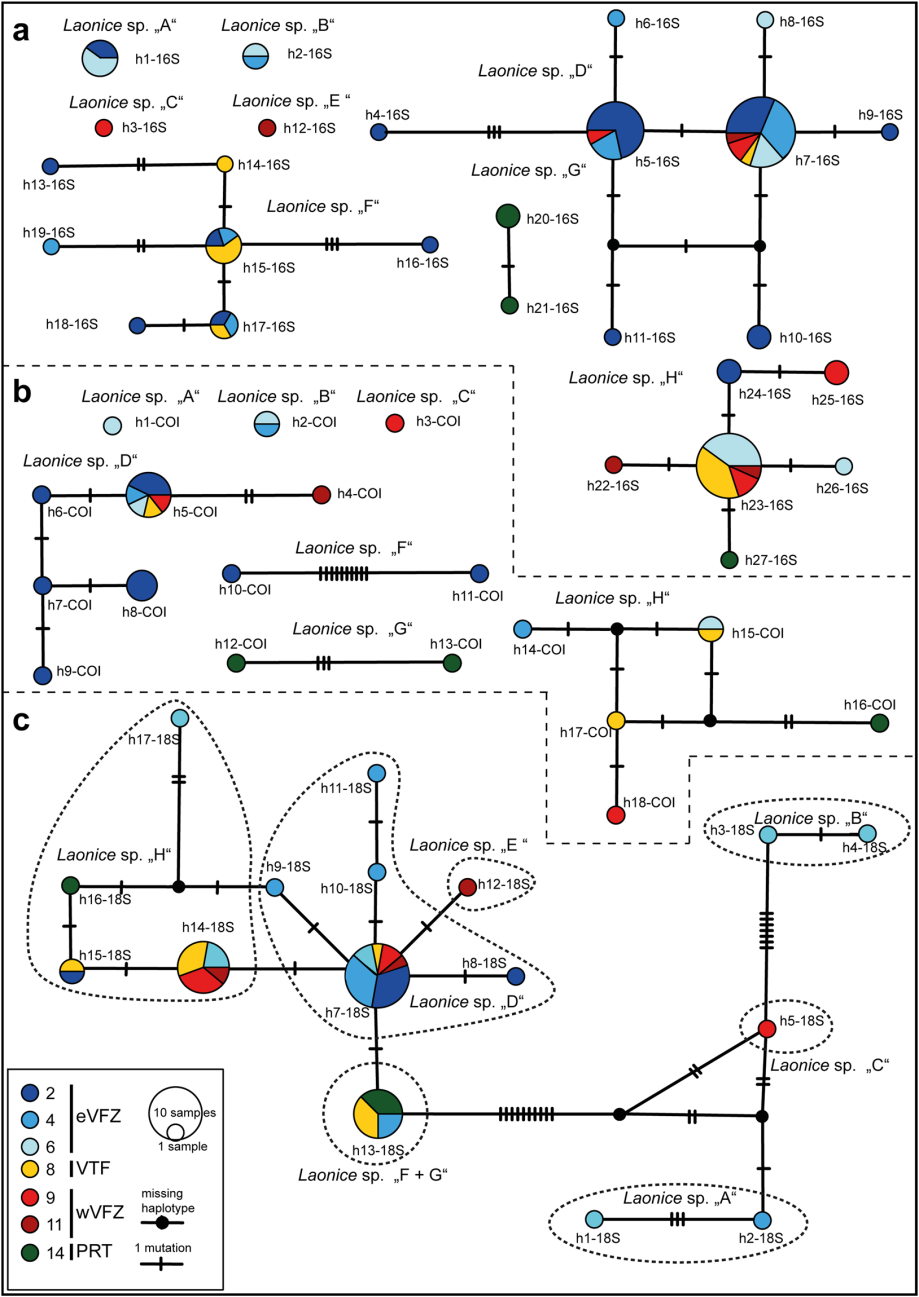
Haplotype networks of Laonice species from the Vema-Transit expedition of 16S (**a**), COI (**b**) and 18S (**c**) gene fragments.

**Figure 4:**
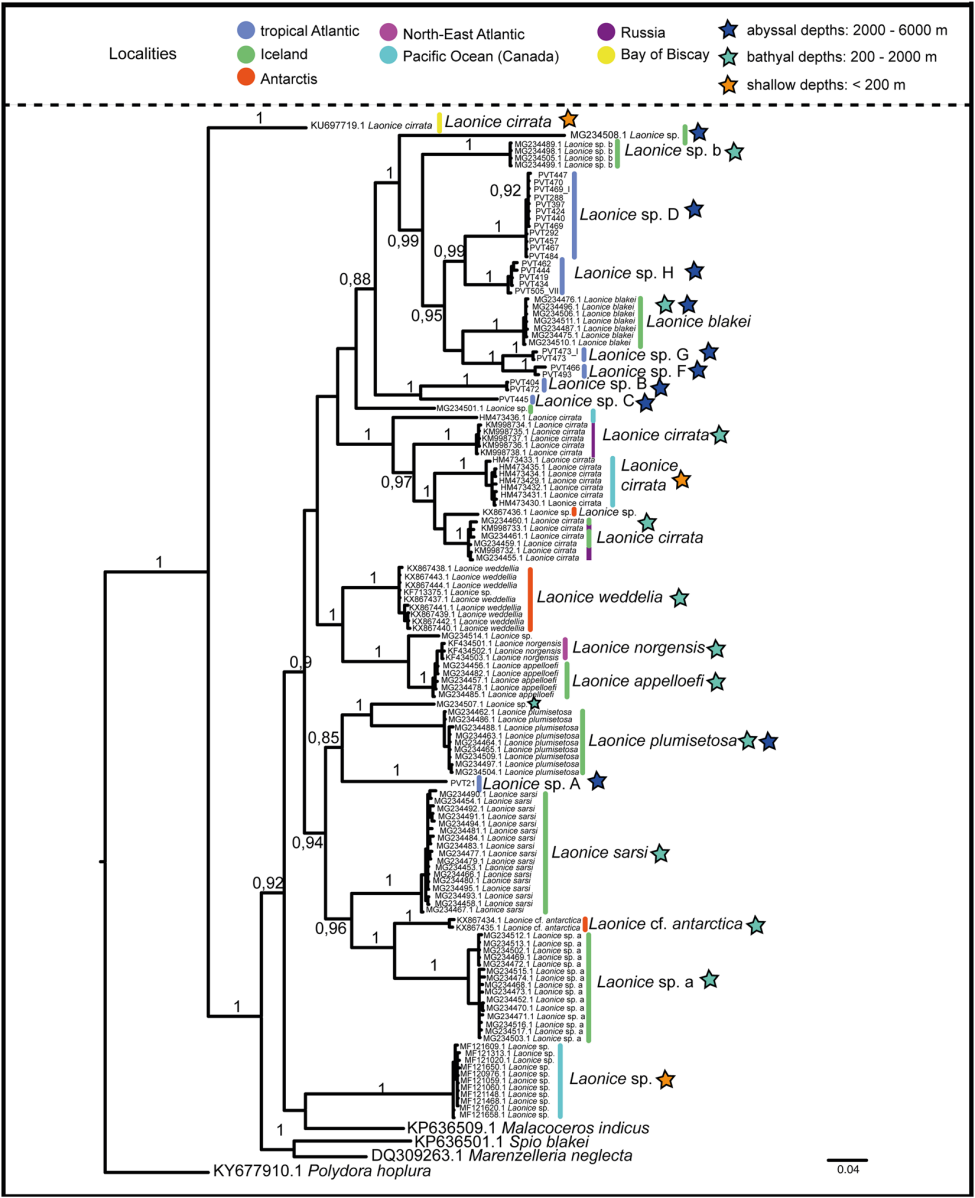
Phylogenetic tree of Laonice specimens from the Atlantic, Antarctic and Pacific Ocean based on the mitochondrial COI gene fragment. Posterior probabilities shown next to the nodes (values below 0.8 are not shown). Sampling localities and depth are colour coded (see legend in upper right-hand corner).

The haplotypes networks of the different gene fragments (16S, COI and 18S) showed slightly different patterns (Fig. 3a–c). For 16S, with the highest number of sequenced individuals, 27 haplotypes (h1-16S-h27-16S) were found with a maximum of eight haplotypes in one lineage (*Laonice* sp. D, Fig. 3a). Networks of COI dataset showed a total of 18 different haplotypes (h1-COI–h18-COI) with a maximum of six haplotypes within the same lineage as in 16S (*Laonice* sp. D, Fig. 4b). For 18S the smallest genetic diversity was found with 17 haplotypes (h1-18S–h17-18S; Fig. 3c). The low number of mutational steps between haplotypes, as evidenced in the 18S network (Fig. 3c), is probably responsible for the lower resolution in the phylogenetic analysis of this gene when it comes to species delimitation. The 18S network shows that *Laonice* sp. A, B and C are well differentiated from each other and the other lineages. *Laonice* sp. D, E, F, G and H all have very similar haplotypes and do not form well differentiated clusters. *Laonice* sp. F and G even share their only haplotype.

As all investigated specimens were incomplete or damaged and relatively short (maximum 22 segments), the main characters for species identification were the shape of the prostomium, the beginning of the lateral pouches, the beginning of the sabre chaeta and the beginning and number of teeth of the neuropodial hooks, as well as the length of the nuchal organ (Table 6).

**Table 6:** Morphological differences (investigated by Guggolz *et al*. 2018^24^) of the eight *Laonice* species (*Laonice* A–G). Question marks are used, if material was insufficient to see characters, respectively.

Species name	No. of spec. characters observed	Nuchal organ end	Start Neuropodial hooks/number of teeth	Lateral pouches start	Sabre chaeta start	Remarks
*Laonice* sp. A	2	end of fragment; 18th chaetiger	9th chaetiger	no pouches seen		prominent dorsolateral ridge 8–11th
*Laonice* sp. B	2	??	??	3rd chaetiger		Peri- und Prostomium fused; very short; 2nd and 3rd branchia different shape than *L*. cf. *blakei*, triangular
*Laonice* sp. C	1	9th chaetiger	??	??	11th chaetiger	Pro-und peristomium not fused; 3–4 rows of cappillaries
*Laonice* sp. D	10	8th − 10th chaetiger	13–15th chaetiger/5 teeth in side view	3rd chaetiger	9th - 11th	no eyes; occipital antennae prominent; Pro-and Peristomium not fused; 3–4 rows of capillaries
*Laonice* sp. E	1	??	16th chaetiger	3rd chaetiger	??	2 rows of capillaries; very short
*Laonice* sp. F	3	9th chaetiger	9th - 10th chaetiger	3rd chaetiger	10th chaetiger	
*Laonice* sp. G	2	8th chaetiger	??	4th chaetiger	8th chaetiger	
*Laonice* sp. H	4	10th chaetiger	14th/15th chaetiger	3rd chaetiger	11th chaetiger	

Slight morphological variations were observed between the eight species delimitated with molecular analyses. For instance, *Laonice* sp. F and sp. G differ in the beginning of the lateral pouches (sp. F: 3^rd^ chaetiger; sp. G: 4^th^ chaetiger), the beginning of the sabre chaeta (sp. F: 10^th^ chaetiger; sp. G: 8^th^ chaetiger) as well as the length of the nuchal organ (sp. F: until 9^th^ chaetiger; sp. G: until 8^th^ chaetiger) (Table 6). Furthermore, *Laonice* sp. B was the only species with the peri- and prostomium fused and in *Laonice* sp. E the beginning of the neuropodial hooks was observed more posteriorly than in all other species (16^th^ chaetiger). *Laonice* sp. A differed from all other species, as the nuchal organ reached the end of the available fragments (until 18^th^ chaetiger) and a prominent dorsolateral ridge was present from chaetiger 8 –11 (Table 6).

### Distribution of species

In the genus-wide phylogenetic analysis of COI with *Laonice* species from the Atlantic, the Southern Ocean, Russian waters and the North-East Pacific, all *Laonice* lineages identified herein were recovered as monophyletic, and none of these seemed to be conspecific with any of the published *Laonice* sequences (Fig. 4). *Laonice* sp. D, F, G and H constitute a monophylum, within a clade including *Laonice blakei* Sikorski and Jirkov in Sikorski *et al*.^46^ and *Laonice* sp. b sensu Bogantes *et al*.^5^, both sampled from Icelandic waters. *Laonice* sp. B and C constitute a monophyletic group that is sister to a large clade of *Laonice* species, including *Laonice* sp. A, from various localities (Fig. 4).

Five of the lineages were only recorded in one of the four areas: *Laonice* sp. A and B in the eVFZ, *Laonice* sp. C and E in the wVFZ and *Laonice* sp. G in the PRT (Fig. 3). These five lineages were relatively rarely collected with three specimens at most (Supplement 1). In contrast, the other three lineages were recorded at larger geographic scales, in either the eVFZ and VTF (*Laonice* sp. F) or even in all four areas (*Laonice* sp. D and H). Even single haplotypes of these lineages exhibited such extensive distributions and were recorded in all of these areas, except PRT (16S: h5, h7, h15, h17, h23; COI: h5, h15; 18S: h7, h13, h14, h15; Fig. 3). For example, *Laonice* sp. D had one haplotype in each of the three studied genes that occurred in the eVFZ, VTF as well as the wVFZ (Fig. 3: h7-16S, h5-COI, h7-18S).

Population differentiation was not significant, neither between different sites, nor between different areas for the three widely distributed lineages *Laonice* sp. D, F and H (Tables 4 and 5).

## Discussion

Employing mitochondrial markers (16S and COI) we were able to identify eight lineages well supported and consistently delimited. Following a strict DNA barcoding approach (*sensu* Hebert *et al*.^47^), these results might easily be interpreted as eight species. However, mitochondrial markers are linked and thus not independently inherited. Therefore, consistency among these markers does not necessarily equate reproductive isolation among the respective lineages^48^. Consistency with other marker types - e.g., nuclear markers or morphology - does offer the possibility to delimit species adequately^49,50^. Taken all data together, lineages A, B, C, D, E and H can be easily delimited as distinct species, even though the differentiation is less pronounced between lineages D, E and H in 18S. The lack of shared haplotypes, despite their sympatric distribution over large geographic scales, is a good indication of reproductive isolation among them. Lineages F and G shared an identical 18S haplotype and also their pairwise uncorrected distances were the lowest for all pairs of lineages (COI: 8.6–8.8%; 16S: 2.8–4.0%). However, a lack of differentiation in 18S may not be surprising for recently diverged species and the levels of differentiation in COI and 16S are comparable to those observed among other polychaete species, which usually exceeded 5–6% for COI^51–54^^,^. Intraspecific distances were always lower than interspecific distances with a maximum of 1.7% within *Laonice* sp. F for 16S and 0.7% within *Laonice* sp. H for COI (Table 3), similar to the 0–2% uncorrected distances found within *Laonice* species from the North-Atlantic^5^. These results could imply thresholds of about 2% for 16S and 2–8% for COI to distinguish between *Laonice* species.

The present molecular study reveals inconsistencies with previous morphology based studies^24^. Guggolz *et al*.^24^ identified six species (aff. *Lindaspio* sp. 1, *Laonice* sp. 1, 4, 5, 6 and *Laonice* cf. *blakei*). The majority of specimens were identified as *L*. cf. *blakei* (about 86.5% of the identified *Laonice* specimens) and the slight variations observed between individuals were interpreted as intraspecific variability within *L*. cf. *blakei*. Of the six species identified based on their morphology by Guggolz *et al*.^24^, only *Laonice* sp. A (aff. *Lindaspio* sp. 1 in Guggolz *et al*.^24^) and sp. C (*Laonice* sp. 6 in Guggolz *et al*.^24^) could be confirmed in our molecular analyses. Specimens identified as *Laonice* cf. *blakei* by Guggolz *et al*.^24^ are here assigned to six different species based on the results from molecular studies: *Laonice* sp. B, D, E, F, G and H. Furthermore, *Laonice* sp. 2, 4, and 5 are all included in *Laonice* sp. D and *Laonice* sp. 1 included in *Laonice* sp. F (see Fig. 2). Most of the disagreement between the morphological study and the present results can be explained as misinterpretations of morphological differences as intraspecific variability rather than interspecific variation. The slight differences observed among individuals identified as *Laonice* cf. *blakei* probably represent interspecific variation between several species of *Laonice*. Taken together with the molecular data, these variations lend additional support for differentiating the eight species identified herein. For instance, *Laonice* sp. F and sp. G, sharing the same 18S haplotype, showed differences in their morphology, supporting a separation at the species level. Comparable morphological differences can be found for *Laonice* sp. A–E as well.

These morphological patterns support the differentiation of the eight lineages and we therefore propose that these eight lineages represent eight species. The lack of differentiation in 18S is probably caused by a combination of a low substitution rate and incomplete lineage sorting^55^ rather than ongoing reproduction among these species.

Apart from delimiting species, we were interested in distribution patterns of the species. Even over large geographic distances (>4,000 km; Table 1, Fig. 3), there seems to be no genetic differentiation within some species. This is most obvious for species distributed across the MAR (*Laonice* sp. D, H), as the same haplotypes are found in the eVFZ and the wVFZ. Species restricted to only one (*Laonice* sp. A and B in the eVFZ) or two of the areas (*Laonice* sp. F in the eVFZ and the VTF) exhibited identical haplotypes across distances of hundreds of kilometres. These species might represent rare species and we could have missed them in the other areas due to the sampling design, as we managed to obtain a higher number of individuals from the eVFZ compared to the other sampled areas^24,25^ (Supplement 1). The present data suggest gene flow over the MAR or potentially through Fracture Zones in the tropical North Atlantic, supported by the low and non-significant levels of differentiation among populations (*Laonice* sp. D, F and H). Guggolz *et al*.^24^ already suggested that the Mid-Atlantic Ridge (MAR) does not represent a physical barrier for some polychaetes based on morphological studies and the lack of significant differences between the eastern and western sides of the ridge. A widespread distribution over 4,000 km was never proven genetically for *Laonice*, but it was reported for other abyssal taxa like *Aurospio dibranchiata* Maciolek, 1981^56^, a polychaete species occurring in different oceans^37^ and *Nicomache lokii* Kongsrud & Rapp, 2012^57^ and *Sclerolinum contortum* Smirnov, 2000^58^, polychaetes living in chemosynthetic-based ecosystems distributed from the Arctic to Antarctic^59^. Larval distribution is suggested to play a major role in the efficiency of the distribution of deep-sea invertebrates, even if the specific larvae are unknown for most species^60^. The exact types of development of the investigated *Laonice* specimens from the tropical North Atlantic is unknown, but in general *Laonice* is supposed to have long-lived larvae and very high dispersal capabilities^12,14,24^. The development strategies seem to be highly connected with the ability to distribute in the abyss even with potential topographic barriers like ridges, rises or canyons. For instance, different molluscs with planktonic larvae were reported to be able to distribute over such barriers^61,62^. Contrary, taxa with direct development, such as brooding isopods, were found to have a restricted distribution with limited or no gene flow across the MAR^18,63^.

None of the eight species recorded in the tropical North Atlantic were found to be conspecific with *Laonice* species for which published genetic data was available. Bogantes *et al*.^5^ recently performed first phylogenetic studies on *Laonice* and suggested that the Antarctic was colonized several times independently. A comparable pattern can be found in our study. Nonetheless, one should keep in mind that these results are based only on one gene (COI) and only a small proportion of known *Laonice* species are included.

Until now, around 16 deep-sea *Laonice* species have been described, mainly based on morphology^9^. Unfortunately, it is not possible to perform subsequent molecular studies with most of the described material, due to fixation, unless new material is collected from the respective type localities. Identification of *Laonice* specimens from deep-sea samples is almost always difficult due to fragmentation and the subsequent loss of important characters independently of the fixation method^5,24^. Therefore, molecular techniques might be of great importance for a correct estimation of their diversity. DNA extraction from fresh material before fixation in formalin takes place would be an appropriate way to combine morphology and molecular studies in soft-bodied animals like spionid polychaetes and should be part of the workflow during sampling.

The present study gives new insights into the phylogeny of *Laonice* and stresses the importance of molecular analyses for estimates of species diversity, ideally combined with morphological studies. The eight *Laonice* species identified in the tropical North Atlantic might be new to science, and certainly do not belong to any of the *Laonice* species investigated with molecular tools to date. Due to the incomplete specimens and thus the absence of important morphological characters, a clear differentiation from all described *Laonice* species is impossible. Therefore, at present the identified lineages cannot be described as new species. However, molecular data is sparse for the genus and new information would further improve our understanding of the evolution of *Laonice* and the dynamics of speciation in the deep-sea. Our present study highlights the importance of integrative taxonomy to allow species delimitation in deep-sea spionids.

The genus’ potential to disperse over large geographic distances in the deep-sea and across topographic barriers such as ridges is shown here and support the hypothesis of other studies^14,64^. We were able to show the occurrence of the same *Laonice* species from the Caribbean to the abyssal plain near West-Africa, highlighting for the first time such a wide distribution for a species of this genus based on molecular analyses. These dispersal abilities are also notable for annelids in general, showing the relevance of molecular tools for our understanding of their distribution in the deep-sea.

## Supplementary information

Supplement 1-3
