# Supplementary material for: Diversity and distribution of Laonice species (Annelida: Spionidae) in the tropical North Atlantic and Puerto Rico Trench
Source: Sci Rep. 2019 Jun 25;9:9260. doi: 10.1038/s41598-019-45807-7 (PMC6592887; doi:10.1038/s41598-019-45807-7)
Supplement: Supplementary file 1 — Supplement 1-3 [file 41598_2019_45807_MOESM1_ESM.pdf]

Diversity and distribution of *Laonice* species (Annelida: Spionidae) in the tropical North Atlantic and Puerto Rico Trench

Theresa Guggolz, Karin Meißner, Martin Schwentner and Angelika Brandt

Supplementary Information

Supplement 1. Specimens code, collection site, haplotype group, lineages/species and GenBank Accession numbers, deposit numbers and collection locations for all *Laonice* specimens from the Vema-Transit expedition of this study.

Specimens code	site	area	Lineage//sp.	location (Lat [start], Long [start] - Lat [end], Long [end])	Haplotype group	depth [m]	Accession no. COI	Accession no. 16S	Accession no. 18S	Deposit No. [ZMH-P]
PVT21	6	eVFZ	A	36°55.06'W, 10°21.82'N - 36°55.06'W, 10°21.83'N	h1-16S, h1-COI, h1-18S	5079	MK507583	---	MK507647	27954
PVT273	2	eVFZ	D	25°03.21' W, 10°42.891'N - 25°03.167'W, 10°42.92'N	h5-16S	5507	---	MK507686	---	28021
PVT280	2	eVFZ	D	25°03.72'W, 10°43.78'W - 25°03.73'W, 10°43.79'N	h5-16S, h7-18S	5520	---	MK507694	MK507629	28051
PVT288	4	eVFZ	D	31°04.40' W, 10°25.62'N - 31°04.37'W, 10°25.62'N	h7-16S, h5-COI, h7-18S	5725	MK507593	MK507703	MK507643	28023
PVT292	4	eVFZ	D	31°04.40' W, 10°25.62'N - 31°04.37'W, 10°25.62'N	h5-16S, h7-COI, h7-18S	5725	MK507589	MK507700	MK507630	28022
PVT292_I	4	eVFZ	D	31°04.40' W, 10°25.62'N - 31°04.37'W, 10°25.62'N	h7-16S, h11-18S	5725	---	MK507695	MK507608	28819
PVT292_II	4	eVFZ	F	31°04.40' W, 10°25.62'N - 31°04.37'W, 10°25.62'N	h16-16S	5725	---	MK507710	---	28833
PVT292_III	4	eVFZ	D	31°04.40' W, 10°25.62'N - 31°04.37'W, 10°25.62'N	h7-16S	5725	---	MK507687	---	28820
PVT379	4	eVFZ	D	31°04.40' W, 10°25.62'N - 31°04.37'W, 10°25.62'N	h6-16S	5725	---	MK507679	---	28041
PVT385_II	4	eVFZ	F	31°02.98'W, 10°25.65'N - 31°02.98'W, 10°25.66'N	h17-16S, h13-18S	5733	---	MK507715	MK507620	28834
PVT385_III	4	eVFZ	D	31°02.98'W, 10°25.65'N - 31°02.98'W, 10°25.66'N	h5-16S	5733	---	MK507680	---	28824
PVT385_IV	4	eVFZ	D	31°02.98'W, 10°25.65'N - 31°02.98'W, 10°25.66'N	h7-16S	5733	---	MK507683	---	28825
PVT385_VI	4	eVFZ	D	31°02.98'W, 10°25.65'N - 31°02.98'W, 10°25.66'N	h7-16S, h7-18S	5733	---	MK507688	MK507631	28826
PVT385_VII	4	eVFZ	D	31°02.98'W, 10°25.65'N - 31°02.98'W, 10°25.66'N	h5-16S, h7-18S	5733	---	MK507659	MK507632	28827
PVT390_I	4	eVFZ	D	31°02.98'W, 10°25.65'N - 31°02.98'W, 10°25.66'N	h7-16S, h9-18S	5733	---	MK507685	MK507645	28828
PVT390_II	4	eVFZ	D	31°02.98'W, 10°25.65'N - 31°02.98'W, 10°25.66'N	h7-16S, h14-18S	5733	---	MK507689	MK507689	28852
PVT390_III	4	eVFZ	F	31°02.98'W, 10°25.65'N - 31°02.98'W, 10°25.66'N	h15-16S, h13-18S	5733	---	MK507717	MK507621	28835
PVT397	6	eVFZ	D	36°55.06'W, 10°21.82'N - 36°55.06'W, 10°21.83'N	h7-16S, h5-COI, h10-18S	5079	MK507594	MK507702	MK507646	28026
PVT397_I	6	eVFZ	H	36°55.06'W, 10°21.82'N - 36°55.06'W, 10°21.83'N	h23-16S	5079	---	MK507666	---	28843
PVT397_II	6	eVFZ	H	36°55.06'W, 10°21.82'N - 36°55.06'W, 10°21.83'N	h23-16S, h14-18S	5079	---	MK507668	MK507607	28844
PVT397_III	6	eVFZ	H	36°55.06'W, 10°21.82'N - 36°55.06'W, 10°21.83'N	h23-16S	5079	---	MK507674	---	28845
PVT404	6	eVFZ	B	36°55.06'W, 10°21.82'N - 36°55.06'W, 10°21.83'N	h2-16S, h2-COI, h4-18S	5079	MK507604	MK507656	MK507649	28098
PVT410	6	eVFZ	D	36°55.35'W, 10°22.65'N - 36°55.35'W, 10°22.66'N	h7-18S	5127	---	---	MK507633	28027
PVT410_I	6	eVFZ	H	36°55.35'W, 10°22.65'N - 36°55.35'W, 10°22.66'N	h26-16S, h14-18S	5127	---	MK507678	MK507609	28846
PVT410_II	6	eVFZ	H	36°55.35'W, 10°22.65'N - 36°55.35'W, 10°22.66'N	h23-16S	5127	---	MK507667	---	28847
PVT410_III	6	eVFZ	H	36°55.35'W, 10°22.65'N - 36°55.35'W, 10°22.66'N	h23-16S	5127	---	MK507670	---	28848
PVT410_IV	6	eVFZ	D	36°55.35'W, 10°22.65'N - 36°55.35'W, 10°22.66'N	h7-16S	5127	---	MK507690	---	28821
PVT412	6	eVFZ	A	36°55.35'W, 10°22.65'N - 36°55.35'W, 10°22.66'N	h1-16S, h2-18S	5127	---	MK507654	MK507648	27955
PVT419	6	eVFZ	H	36°55.35'W, 10°22.65'N - 36°55.35'W, 10°22.66'N	h23-16S, h15-COI	5127	MK507600	MK507662	---	28029
PVT419_I	6	eVFZ	D	36°55.35'W, 10°22.65'N - 36°55.35'W, 10°22.66'N	h7-16S, h7-18S	5127	---	MK507691	MK507634	28822
PVT424	8	VTF	D	42°39.73'W, 10°43.00'N - 42°39.73'W, 10°43.00'N	h7-16S, h5-COI, h7-18S	5178	MK507595	MK507704	MK507635	28031
PVT424_I	8	VTF	H	42°39.73'W, 10°43.00'N - 42°39.73'W, 10°43.00'N	h23-16S, h14-18S	5178	---	MK507663	MK507610	28837
PVT424_II	8	VTF	F	42°39.73'W, 10°43.00'N - 42°39.73'W, 10°43.00'N	h14-16S, h13-18S	5178	---	MK507712	MK507622	28829
PVT424_III	8	VTF	F	42°39.73'W, 10°43.00'N - 42°39.73'W, 10°43.00'N	h17-16S	5178	---	MK507705	---	28830
PVT424_IV	8	VTF	H	42°39.73'W, 10°43.00'N - 42°39.73'W, 10°43.00'N	h23-16S	5178	---	MK507661	---	28838
PVT424_V	8	VTF	F	42°39.73'W, 10°43.00'N - 42°39.73'W, 10°43.00'N	h15-16S, h13-18S	5178	---	MK507718	MK507623	28831
PVT424_VII	8	VTF	H	42°39.73'W, 10°43.00'N - 42°39.73'W, 10°43.00'N	h15-16S, h14-18S	5178	---	MK507713	MK507611	28839
PVT424_VIII	8	VTF	H	42°39.73'W, 10°43.00'N - 42°39.73'W, 10°43.00'N	h23-16S	5178	---	---	---	28840
PVT424_XI	8	VTF	H	42°39.73'W, 10°43.00'N - 42°39.73'W, 10°43.00'N	h23-16S, h14-18S	5178	---	MK507676	MK507612	28841
PVT424_XII	8	VTF	H	42°39.73'W, 10°43.00'N - 42°39.73'W, 10°43.00'N	h23-16S	5178	---	MK507677	---	28842
PVT434	8	VTF	H	42°39.73'W, 10°43.00'N - 42°39.73'W, 10°43.00'N	h23-16S, h15-COI, h15-18S	5178	MK507601	MK507672	MK507618	28030
PVT440	9	wVFZ	D	47°53.99'W, 11°39.36' N - 47°53.97'W, 11°39.36'N	h7-16S, h5-COI	5001	MK507596	MK507697	---	28034
PVT440_I	9	wVFZ	D	47°53.99'W, 11°39.36' N - 47°53.97'W, 11°39.36'N	h5-16S, h7-18S	5001	---	MK507692	MK507636	28823
PVT440_II	9	wVFZ	H	47°53.99'W, 11°39.36' N - 47°53.97'W, 11°39.36'N	h25-16S, h14-18S	5001	---	MK507670	MK507613	28849
PVT440_III	9	wVFZ	H	47°53.99'W, 11°39.36' N - 47°53.97'W, 11°39.36'N	h23-16S, h14-18S	5001	---	MK507673	---	28850
PVT440_IV	9	wVFZ	H	47°53.99'W, 11°39.36' N - 47°53.97'W, 11°39.36'N	h25-16S, h14-18S	5001	---	MK507671	MK507614	28851
PVT444	9	wVFZ	H	47°53.99'W, 11°39.36' N - 47°53.97'W, 11°39.36'N	h18-COI, h14-18S	5001	MK507602	---	MK507615	28035
PVT445	9	wVFZ	C	47°58.03'W, 11°40.73'N - 47°59.00'W, 11°40.45N	h3-16S, h3-COI, h5-18S	4986	MK507606	MK507658	MK507650	28046
PVT446	9	wVFZ	D	47°58.03'W, 11°40.73'N - 47°59.00'W, 11°40.45N	h7-16S, h7-18S	4986	---	MK507698	MK507637	28045

PVT447	11	wVFZ	D	50°27.97'W, 12°05.84'N - 50°27.96'W, 12°05.81'N	h7-16S, h4-COI, h7-18S	5088	MK507588	MK507723	MK507638	28036
PVT451	11	wVFZ	H	50°28.14'W, 12°04.83'N - 50°28.14' W, 12°04.82'N	h23-16S, h14-18S	5108	---	MK507675	MK507616	28038
PVT 447_I	11	wVFZ	E	50°27.97'W, 12°05.84'N - 50°27.96'W, 12°05.81'N	H12-16S, h12-18S	5088	---	MK507706	MK507644	28814
PVT457	2	eVFZ	D	25°03.21' W, 10°42.891'N - 25°03.167'W, 10°42.92'N	h11-16S, h9-COI	5507	MK507590	MK507722	---	28020
PVT457_I	2	eVFZ	D	25°03.21' W, 10°42.891'N - 25°03.167'W, 10°42.92'N	h5-16S, h7-18S	5507	---	MK507682	MK507639	28816
PVT458	2	eVFZ	D	25°03.21' W, 10°42.891'N - 25°03.167'W, 10°42.92'N	h5-16S	5507	---	MK507699	---	28019
PVT462	14	PRT	H	67°09.247'W, 19°02.097'N - 67°09.43'W, 19°02.11'N	h27-16S, h16-COI, h16-18S	4552	MK507599	MK507720	MK507617	28039
PVT463	14	PRT	G	67°09.247'W, 19°02.097'N - 67°09.43'W, 19°02.11'N	h21-16S, h13-18S	4552	---	MK507708	MK507624	28042
PVT466	2	eVFZ	F	25°03.21' W, 10°42.891'N - 25°03.167'W, 10°42.92'N	h18-16S, h10-COI	5507	MK507586	MK507711	---	28011
PVT467	2	eVFZ	D	25°03.21' W, 10°42.891'N - 25°03.167'W, 10°42.92'N	h5-16S, h8-COI, h7-18S	5507	MK507591	MK507693	MK507640	28014
PVT469	2	eVFZ	A	25°03.21' W, 10°42.891'N - 25°03.167'W, 10°42.92'N	h1-16S	5507	---	MK507655	---	28013
PVT469_I	2	eVFZ	D	25°03.21' W, 10°42.891'N - 25°03.167'W, 10°42.92'N	h4-16S, h5-COI	5507	MK507598	---	---	28817
PVT470	2	eVFZ	D	25°03.72'W, 10°43.78'W - 25°03.73'W, 10°43.79'N	h5-16S, h6-COI, h7-18S	5520	MK507597	MK507721	MK507641	28015
PVT472	4	eVFZ	B	31°02.98'W, 10°25.65'N - 31°02.98'W, 10°25.66'N	h2-16S, h2-COI, h3-18S	5733	MK507605	MK507657	MK507651	28043
PVT473	14	PRT	G	67°09.247'W, 19°02.097'N - 67°09.43'W, 19°02.11'N	h20-16S, h12-COI, h13-18S	4552	MK507585	MK507709	MK507625	28040
PVT473_I	14	PRT	G	67°09.247'W, 19°02.097'N - 67°09.43'W, 19°02.11'N	h20-16S, h13-COI, h13-18S	4552	MK507584	MK507719	MK507626	28836
PVT479	11	wVFZ	H	50°27.97'W, 12°05.84'N - 50°27.96'W, 12°05.81'N	h22-16S	5088	---	MK507664	---	28037
PVT484	2	eVFZ	D	25°03.21' W, 10°42.891'N - 25°03.167'W, 10°42.92'N	h5-16S, h8-18S	5507	---	MK507701	MK507592	28018
PVT491	2	eVFZ	F	25°03.72'W, 10°43.78'W - 25°03.73'W, 10°43.79'N	h17-16S	5520	---	MK507707	---	28017
PVT493	2	eVFZ	F	25°03.72'W, 10°43.78'W - 25°03.73'W, 10°43.79'N	h13-16S, h11-COI	5520	MK507587	---	---	28054
PVT495	2	eVFZ	H	25°03.72'W, 10°43.78'W - 25°03.73'W, 10°43.79'N	h24-16S	5520	---	MK507660	---	28003
PVT495_I	2	eVFZ	F	25°03.72'W, 10°43.78'W - 25°03.73'W, 10°43.79'N	h15-16S	5520	---	MK507716	---	28832
PVT495_II	2	eVFZ	D	25°03.72'W, 10°43.78'W - 25°03.73'W, 10°43.79'N	h7-16S, h7-18S	5520	---	MK507696	MK507642	28815
PVT572_II	2	eVFZ	H	25°03.21' W, 10°42.891'N - 25°03.167'W, 10°42.92'N	h24-16S, h15-18S	5507	---	MK507665	MK507619	28076
PVT592_I	2	eVFZ	D	25°03.21' W, 10°42.891'N - 25°03.167'W, 10°42.92'N	h10-16S	5507	---	MK507652	---	29015
PVT592_II	2	eVFZ	D	25°03.21' W, 10°42.891'N - 25°03.167'W, 10°42.92'N	h5-16S	5507	---	MK507681	---	28807
PVT592_III	2	eVFZ	D	25°03.21' W, 10°42.891'N - 25°03.167'W, 10°42.92'N	h7-16S	5507	---	MK507684	---	28818
PVT921	8	VTF	F	42°39.73'W, 10°43.00'N - 42°39.73'W, 10°43.00'N	h15-16S, h13-18S	5178	---	MK507714	MK507627	28033

Supplement 2. List of all *Laonice* specimens additionally included in this study with GenBank accession numbers and collection locality details.

Species Name	Genbank Number	Location	Latitude	Longitude	Depth [m]	Gene	Deposit Number	Reference
Laonice sp.	MG234507	Iceland	62.55	-20.38	1386.80	COI	DZMB-HH-57470	Bogantes et al. 2018
Laonice sp.	MG234508	Iceland	67.59	-6.96	2402.00	COI	DZMB-HH-57467	Bogantes et al. 2018
Laonice sp.	MG234514	Iceland	61.71	-19.55	1912.30	COI	DZMB-HH-57472	Bogantes et al. 2018
Laonice appelloefi	MG234457	Iceland	62.00	0.51	302.50	COI	DZMB-HH-43976	Bogantes et al. 2018
Laonice appelloefi	MG234485	Iceland	62.00	0.51	302.50	COI	DZMB-HH-37741	Bogantes et al. 2018
Laonice appelloefi	MG234456	Iceland	62.00	0.51	302.50	COI	DZMB-HH-37741	Bogantes et al. 2018
Laonice appelloefi	MG234482	Iceland	62.00	0.51	302.50	COI	DZMB-HH-37741	Bogantes et al. 2018
Laonice appelloefi	MG234478	Iceland	62.00	0.51	302.50	COI	DZMB-HH-47872	Bogantes et al. 2018
Laonice blakei	MG234487	Iceland	60.36	-18.14	2567.60	COI	DZMB-HH-31228	Bogantes et al. 2018
Laonice blakei	MG234476	Iceland	60.36	-18.14	2567.60	COI	DZMB-HH-31228	Bogantes et al. 2018
Laonice blakei	MG234475	Iceland	60.36	-18.14	2567.60	COI	DZMB-HH-31228	Bogantes et al. 2018
Laonice blakei	MG234496	Iceland	62.94	-20.74	913.60	COI	DZMB-HH-57473	Bogantes et al. 2018
Laonice blakei	MG234506	Iceland	62.55	-20.38	1386.80	COI	DZMB-HH-57470	Bogantes et al. 2018
Laonice blakei	MG234510	Iceland	60.36	-18.14	2567.60	COI	DZMB-HH-31228	Bogantes et al. 2018
Laonice blakei	MG234511	Iceland	61.71	-19.55	1912.30	COI	DZMB-HH-57472	Bogantes et al. 2018
Laonice cirrata	MG234461	Iceland	63.42	-10.97	440.50	COI	DZMB-HH-42927	Bogantes et al. 2018
Laonice cirrata	MG234460	Iceland	63.42	-10.97	440.50	COI	DZMB-HH-42927	Bogantes et al. 2018
Laonice cirrata	MG234459	Iceland	63.61	-7.75	1056.20	COI	DZMB-HH-41682	Bogantes et al. 2018
Laonice cirrata	MG234455	Iceland	63.58	-7.71	1043.60	COI	DZMB-HH-40702	Bogantes et al. 2018
Laonice cirrata	MG234485	Iceland	62.99	-28.10	1588.20	COI	DZMB-HH-57475	Bogantes et al. 2018
Laonice cirrata	MG234501	Iceland	62.99	-28.10	1588.20	COI	DZMB-HH-57475	Bogantes et al. 2018
Laonice plumisetosa	MG234465	Iceland	67.64	-12.16	1819.30	COI	SMF-24376	Bogantes et al. 2018
Laonice plumisetosa	MG234464	Iceland	67.64	-12.16	1819.30	COI	SMF-24377	Bogantes et al. 2018
Laonice plumisetosa	MG234463	Iceland	67.64	-12.16	1819.30	COI	SMF-24377	Bogantes et al. 2018
Laonice plumisetosa	MG234486	Iceland	67.64	-12.16	1819.30	COI	SMF-24377	Bogantes et al. 2018
Laonice plumisetosa	MG234462	Iceland	69.09	-9.93	2173.40	COI	DZMB-HH-34036	Bogantes et al. 2018
Laonice plumisetosa	MG234488	Iceland	67.59	-6.96	2402.00	COI	DZMB-HH-57466	Bogantes et al. 2018
Laonice plumisetosa	MG234497	Iceland	62.94	-20.74	913.60	COI	DZMB-HH-57473	Bogantes et al. 2018
Laonice plumisetosa	MG234504	Iceland	62.94	-20.74	913.60	COI	DZMB-HH-57465	Bogantes et al. 2018

Laonice plumisetosa	MG234509	Iceland	67.59	-6.96	2402.00	COI	SMF-24379	Bogantes et al. 2018
Laonice sarsi	MG234467	Iceland	63.31	-23.16	288.50	COI	DZMB-HH-37273	Bogantes et al. 2018
Laonice sarsi	MG234466	Iceland	63.31	-23.16	288.50	COI	DZMB-HH-37273	Bogantes et al. 2018
Laonice sarsi	MG234458	Iceland	62.00	0.51	302.50	COI	DZMB-HH-43976	Bogantes et al. 2018
Laonice sarsi	MG234484	Iceland	62.00	0.51	302.50	COI	DZMB-HH-37741	Bogantes et al. 2018
Laonice sarsi	MG234483	Iceland	62.00	0.51	302.50	COI	DZMB-HH-37741	Bogantes et al. 2018
Laonice sarsi	MG234454	Iceland	62.00	0.51	302.50	COI	DZMB-HH-47872	Bogantes et al. 2018
Laonice sarsi	MG234481	Iceland	62.00	0.51	302.50	COI	DZMB-HH-47872	Bogantes et al. 2018
Laonice sarsi	MG234453	Iceland	62.00	0.51	302.50	COI	DZMB-HH-47872	Bogantes et al. 2018
Laonice sarsi	MG234480	Iceland	62.00	0.51	302.50	COI	DZMB-HH-47872	Bogantes et al. 2018
Laonice sarsi	MG234479	Iceland	62.00	0.51	302.50	COI	DZMB-HH-47872	Bogantes et al. 2018
Laonice sarsi	MG234477	Iceland	62.00	0.51	302.50	COI	DZMB-HH-47872	Bogantes et al. 2018
Laonice sarsi	MG234490	Iceland	63.31	-23.16	288.50	COI	DZMB-HH-57474	Bogantes et al. 2018
Laonice sarsi	MG234491	Iceland	63.31	-23.16	288.50	COI	DZMB-HH-57474	Bogantes et al. 2018
Laonice sarsi	MG234492	Iceland	63.31	-23.16	288.50	COI	DZMB-HH-57474	Bogantes et al. 2018
Laonice sarsi	MG234493	Iceland	63.31	-23.16	288.50	COI	DZMB-HH-57474	Bogantes et al. 2018
Laonice sarsi	MG234494	Iceland	63.31	-23.16	288.50	COI	DZMB-HH-57474	Bogantes et al. 2018
Laonice sarsi	MG234495	Iceland	63.31	-23.16	288.50	COI	DZMB-HH-57474	Bogantes et al. 2018
Laonice sp. a	MG234474	Iceland	61.71	-19.55	1912.30	COI	DZMB-HH-20413	Bogantes et al. 2018
Laonice sp. a	MG234473	Iceland	61.71	-19.55	1912.30	COI	DZMB-HH-20414	Bogantes et al. 2018
Laonice sp. a	MG234472	Iceland	61.71	-19.55	1912.30	COI	DZMB-HH-20415	Bogantes et al. 2018
Laonice sp. a	MG234471	Iceland	61.71	-19.55	1912.30	COI	DZMB-HH-20417	Bogantes et al. 2018
Laonice sp. a	MG234470	Iceland	61.71	-19.55	1912.30	COI	DZMB-HH-20418	Bogantes et al. 2018
Laonice sp. a	MG234469	Iceland	61.71	-19.55	1912.30	COI	DZMB-HH-20419	Bogantes et al. 2018
Laonice sp. a	MG234468	Iceland	61.71	-19.55	1912.30	COI	DZMB-HH-20427	Bogantes et al. 2018
Laonice sp. a	MG234452	Iceland	62.55	-20.40	1384.80	COI	DZMB-HH-32623	Bogantes et al. 2018
Laonice sp. a	MG234502	Iceland	62.99	-28.10	1588.20	COI	DZMB-HH-57475	Bogantes et al. 2018
Laonice sp. a	MG234503	Iceland	62.55	-20.40	1384.80	COI	DZMB-HH-32623	Bogantes et al. 2018
Laonice sp. a	MG234512	Iceland	61.71	-19.55	1912.30	COI	DZMB-HH-57472	Bogantes et al. 2018
Laonice sp. a	MG234513	Iceland	61.71	-19.55	1912.30	COI	DZMB-HH-57472	Bogantes et al. 2018
Laonice sp. a	MG234515	Iceland	61.71	-19.55	1912.30	COI	DZMB-HH-57472	Bogantes et al. 2018
Laonice sp. a	MG234516	Iceland	61.71	-19.55	1912.30	COI	DZMB-HH-57472	Bogantes et al. 2018
Laonice sp. a	MG234517	Iceland	61.71	-19.55	1912.30	COI	DZMB-HH-57472	Bogantes et al. 2018

Laonice sp. b	MG234489	Iceland	62.93	-20.77	891.70	COI	DZMB-HH-57471	Bogantes et al. 2018
Laonice sp. b	MG234498	Iceland	62.94	-20.74	913.60	COI	DZMB-HH-57473	Bogantes et al. 2018
Laonice sp. b	MG234499	Iceland	62.94	-20.74	913.60	COI	DZMB-HH-57473	Bogantes et al. 2018
Laonice sp. b	MG234505	Iceland	62.94	-20.74	913.60	COI	DZMB-HH-57465	Bogantes et al. 2018
Laonice cf. *antarctica*	KX867434.1	Antarctica	---	---	100-3500	COI	---	Brasier et al. 2017
Laonice cf. *antarctica*	KX867435.1	Antarctica	---	---	100-3500	COI	---	Brasier et al. 2017
Laonice cirrata	HM473429.1	British Columbia, Canada	48.89	-125.18	17.00	COI	BAMPOL0239	Carr et al. 2011
Laonice cirrata	HM473430.1	British Columbia, Canada	48.89	-125.18	17.00	COI	BAMPOL0247	Carr et al. 2011
Laonice cirrata	HM473431.1	British Columbia, Canada	48.89	-125.18	17.00	COI	BAMPOL0256	Carr et al. 2011
Laonice cirrata	HM473432.1	British Columbia, Canada	48.89	-125.18	17.00	COI	BAMPOL0274	Carr et al. 2011
Laonice cirrata	HM473433.1	British Columbia, Canada	48.89	-125.18	17.00	COI	BAMPOL0280	Carr et al. 2011
Laonice cirrata	HM473434.1	British Columbia, Canada	48.89	-125.18	17.00	COI	BAMPOL0282	Carr et al. 2011
Laonice cirrata	HM473435.1	British Columbia, Canada	48.89	-125.18	17.00	COI	BAMPOL0284	Carr et al. 2011
Laonice cirrata	HM473436.1	British Columbia, Canada	48.89	-125.18	17.00	COI	BAMPOL0284	Carr et al. 2011
Laonice cirrata	KM998732.1	Russia	---	---	---	COI	---	Radashevsky unpublished
Laonice cirrata	KM998733.1	Russia	---	---	---	COI	---	Radashevsky unpublished
Laonice cirrata	KM998734.1	Russia	---	---	---	COI	---	Radashevsky unpublished
Laonice cirrata	KM998735.1	Russia	---	---	---	COI	---	Radashevsky unpublished
Laonice cirrata	KM998736.1	Russia	---	---	---	COI	---	Radashevsky unpublished
Laonice cirrata	KM998737.1	Russia	---	---	---	COI	---	Radashevsky unpublished
Laonice cirrata	KM998738.1	Russia	---	---	---	COI	---	Radashevsky unpublished
Laonice cirrata	KX867436.1	Antarctica	---	---	100-3500	COI	---	Brasier et al. 2017
Laonice norgensis	KF434501.1	NE Atlantic	29.74	-28.41	285.50	COI	ZSRO-P2293	Meißner et al. 2014
Laonice norgensis	KF434502.1	NE Atlantic	29.60	-28.98	268.20	COI	ZSRO-P2292	Meißner et al. 2014
Laonice norgensis	KF434503.1	NE Atlantic	29.74	-28.42	283.30	COI	ZSRO-P2295	Meißner et al. 2014
Laonice sp.	KF713375.1	Southern Ocean	-66.94	170.85	451.00	COI	---	Gallego et al. 2015
Laonice weddellia	KX867437.1	Southern Ocean	---	---	100-3500	COI	---	Brasier et al. 2017
Laonice weddellia	KX867438.1	Southern Ocean	---	---	100-3500	COI	---	Brasier et al. 2017
Laonice weddellia	KX867439.1	Southern Ocean	---	---	100-3500	COI	---	Brasier et al. 2017
Laonice weddellia	KX867440.1	Southern Ocean	---	---	100-3500	COI	---	Brasier et al. 2017
Laonice weddellia	KX867441.1	Southern Ocean	---	---	100-3500	COI	---	Brasier et al. 2017
Laonice weddellia	KX867442.1	Southern Ocean	---	---	100-3500	COI	---	Brasier et al. 2017
Laonice weddellia	KX867443.1	Southern Ocean	---	---	100-3500	COI	---	Brasier et al. 2017

Laonice weddellia	KX867444.1	Southern Ocean	---	---	100-3500	COI	---	Brasier et al. 2017
Laonice sp.	KX867436.1	Southern Ocean	---	---	100-3500	COI	---	Brasier et al. 2017
Laonice sp.	DQ779619.1	Sweden	---	---	---	16S	---	Rousset et al. 2007
Laonice sp.	KX867291.1	Antarctica	---	---	100-3500	16S	---	Brasier et al. 2017
Laonice sp.	KX867290.1	Antarctica	---	---	100-3500	16S	---	Brasier et al. 2017
Laonice norgensis	KF434512.1	NE Atlantic	29.60	-28.98	268.20	16S	ZSRO-P2292	Meißner et al. 2014
Laonice norgensis	KX867304.1	Antarctica	---	---	100-3500	16S	---	Brasier et al. 2017
Laonice weddellia	KX867307.1	Antarctica	---	---	100-3500	16S	---	Brasier et al. 2017
Laonice weddellia	KX867311.1	Antarctica	---	---	100-3500	16S	---	Brasier et al. 2017
Laonice weddellia	KX867305.1	Antarctica	---	---	100-3500	16S	---	Brasier et al. 2017
Laonice weddellia	KX867297.1	Antarctica	---	---	100-3500	16S	---	Brasier et al. 2017
Laonice weddellia	KX867310.1	Antarctica	---	---	100-3500	16S	---	Brasier et al. 2017
Laonice weddellia	KX867295.1	Antarctica	---	---	100-3500	16S	---	Brasier et al. 2017
Laonice weddellia	KT383399.1	Southern Ocean	-66.94	170.85	451.00	16S	---	Gallego et al. 2015
Laonice weddellia	KX867294.1	Antarctica	---	---	100-3500	16S	---	Brasier et al. 2017
Laonice weddellia	KX867303.1	Antarctica	---	---	100-3500	16S	---	Brasier et al. 2017
Laonice weddellia	KX867312.1	Antarctica	---	---	100-3500	16S	---	Brasier et al. 2017
Laonice weddellia	KX867296.1	Antarctica	---	---	100-3500	16S	---	Brasier et al. 2017
Laonice weddellia	KX867302.1	Antarctica	---	---	100-3500	16S	---	Brasier et al. 2017
Laonice weddellia	KX867313.1	Antarctica	---	---	100-3500	16S	---	Brasier et al. 2017
Laonice weddellia	KX867300.1	Antarctica	---	---	100-3500	16S	---	Brasier et al. 2017
Laonice weddellia	KX867293.1	Antarctica	---	---	100-3500	16S	---	Brasier et al. 2017
Laonice weddellia	KX867301.1	Antarctica	---	---	100-3500	16S	---	Brasier et al. 2017
Laonice weddellia	KX867309.1	Antarctica	---	---	100-3500	16S	---	Brasier et al. 2017
Laonice weddellia	KX867299.1	Antarctica	---	---	100-3500	16S	---	Brasier et al. 2017
Laonice weddellia	KF713471.1	Southern Ocean	-66.94	170.85	451.00	16S	---	Gallego et al. 2015
Laonice weddellia	KX867314.1	Antarctica	---	---	100-3500	16S	---	Brasier et al. 2017
Laonice weddellia	KX867292.1	Antarctica	---	---	100-3500	16S	---	Brasier et al. 2017
Laonice weddellia	KX867298.1	Antarctica	---	---	100-3500	16S	---	Brasier et al. 2017
Laonice weddellia	KX867308.1	Antarctica	---	---	100-3500	16S	---	Brasier et al. 2017
Laonice cf. *vieitzi*	KX867285.1	Antarctica	---	---	100-3500	16S	---	Brasier et al. 2017
Laonice cf. *vieitzi*	KX867286.1	Antarctica	---	---	100-3500	16S	---	Brasier et al. 2017
Laonice cf. *vieitzi*	KX867287.1	Antarctica	---	---	100-3500	16S	---	Brasier et al. 2017

Laonice cf. *vieitzi*	KX867288.1	Antarctica	---	---	100-3500	16S	---	Brasier et al. 2017
Laonice cf. *vieitzi*	KX867289.1	Antarctica	---	---	100-3500	16S	---	Brasier et al. 2017
Laonice sp.	EU340088.1	whale fall／California borderland basins, USA	---	---	---	16S	---	Mincks et al. 2009
Laonice cf. *antarctica*	KX867283.1	Antarctica	---	---	100-3500	16S	---	Brasier et al. 2017
Laonice cf. *antarctica*	KX867280.1	Antarctica	---	---	100-3500	16S	---	Brasier et al. 2017
Laonice cf. *antarctica*	KF713472.1	Southern Ocean	-66.94	170.85	451.00	16S	---	Gallego et al. 2015
Laonice cf. *antarctica*	KX867281.1	Antarctica	---	---	100-3500	16S	---	Brasier et al. 2017
Laonice cf. *antarctica*	KX867284.1	Antarctica	---	---	100-3500	16S	---	Brasier et al. 2017
Laonice cf. *antarctica*	KT383400.1	Southern Ocean	-66.94	170.85	451.00	16S	---	Gallego et al. 2015
Laonice cf. *antarctica*	KX867279.1	Antarctica	---	---	100-3500	16S	---	Brasier et al. 2017
Laonice cf. *antarctica*	KX867282.1	Antarctica	---	---	100-3500	16S	---	Brasier et al. 2017
Polydora hoplura	LC101881.1	Saldanha, South Africa	---	---	shallow water	16S	---	Sato-Okoshi et al., 2016
Polydora hoplura	KY677910.1	Saldanha, South Africa	---	---	shallow water	COI	---	Williams, L.-G. et al., 2017
Spio blakei	KP636501.1	Lizard Island, Queensland, Australia	-14.68	145.45	0.50	COI	AM W44372	Meißner and Götting, 2015
Spio blakei	KP636502.1	Lizard Island, Queensland, Australia	-14.68	145.45	0.50	16S	AM W44372	Meißner and Götting, 2015
Marenzelleria neglecta	DQ309254.1	Elbe river estuary	---	---	---	16S	---	Bastrop & Blank, 2006
Marenzelleria neglecta	DQ309263.1	Elbe river estuary	---	---	---	COI	---	Bastrop & Blank, 2006
Malacoceros indicus	KP636510.1	Lizard Island, Queensland, Australia	-14.68	145.45	0.50	16S	AM W44378	Meißner and Götting, 2015
Malacoceros indicus	KP636509.1	Lizard Island, Queensland, Australia	-14.68	145.45	0.50	16S	AM W44378	Meißner and Götting, 2015

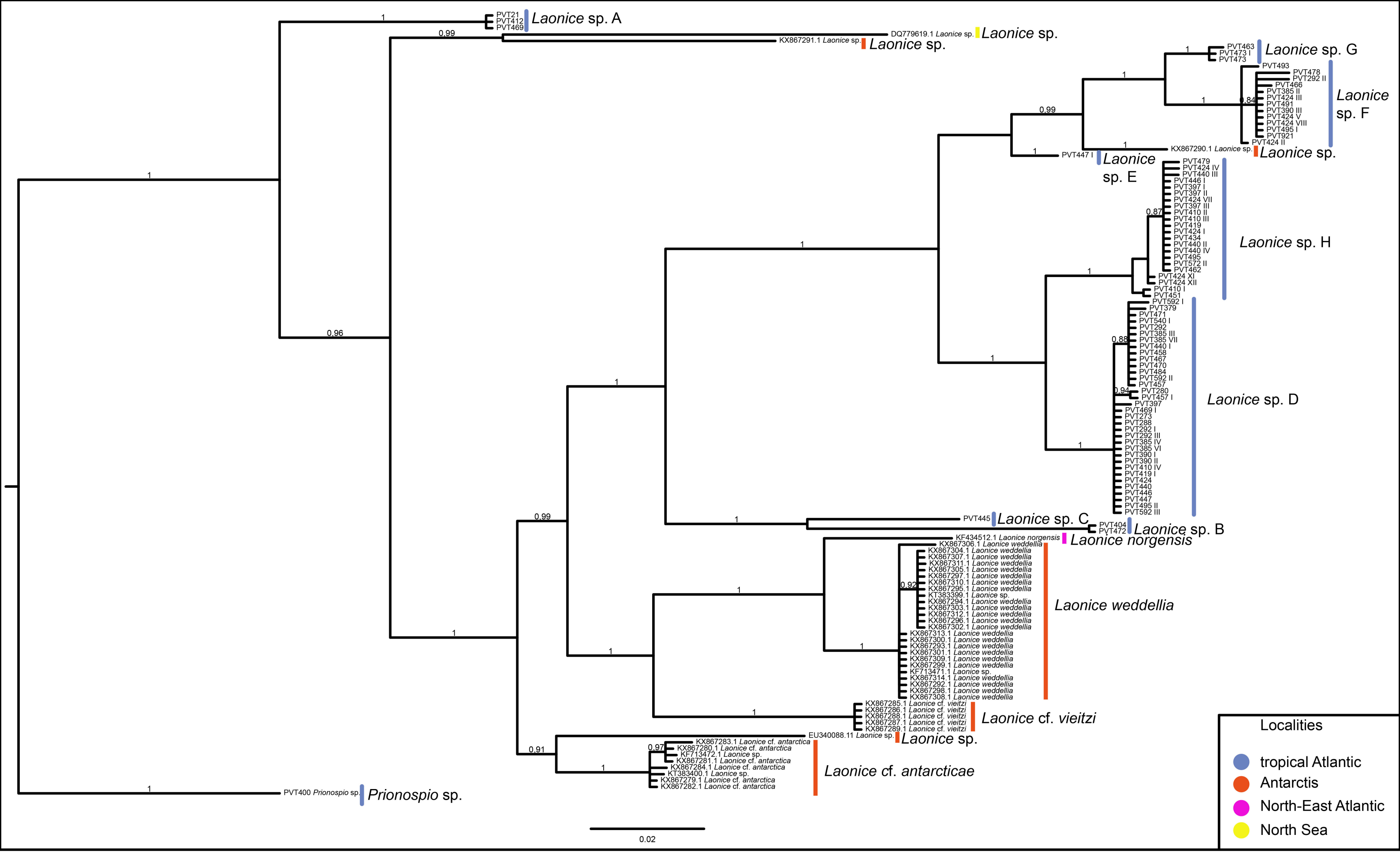

Supplement 3. Phylogenetic tree of Laonice specimens from the Atlantic, the Ross Sea and the Southern Ocean based on the mitochondrial 16S gene fragment. Posterior probabilities shown next to the nodes (values below 0.8 are not shown). Sampling localities are colour coded. Different depths are marked with stars (abyssal: blue; bathyal: turquoise; shallow: orange).
